# A Paper Read before the Montgomery County Medical Society, Ill. June, 1873

**Published:** 1873-09

**Authors:** T. D. Washburn


BUFFALO

Medical and Surgical Journal.

VOL XIII. SEPTEMBER, 1873. No. 2.

### Original Communications.

ART. I—*A Paper read before the Montgomery Co. Medical Society.* Illinois, June 1873. By T. D. WASHBURN, M. D.

The President of the last National Medical Association in St. Louis, in his address uses the following language:

"Let us throw away all puerile notions about the dignity of our calling and approach the people through the only channels by which they can be reached—the newspaper and the Lecture-room. This is our work for the future—to educate the people."

Again he says after giving them to understand, he does not wish to do more than "spread abroad such sound ideas of enlightened hygiene as will enable the people to co-operate with us in correcting all these formidable obliquities—physical, mental and moral, which are insidiously polluting the stream of humanity."

"Such publications as your president now proposes cannot be misconstrued—they cannot be tortured into a violation of the code," and then adds if they can let us alter or amend the code, etc. These are *wise* and most *timely* words: coming from the most representative body and a representative man, well should they be heeded.

Has not the time arrived, when we should make an advance in this direction? Can we afford to let our enemies occupy this vantage ground, and bring their batteries and small arms to bear on us, and we remain silent?

How is the public mind in regard to the great preacher of the nineteenth century, upon whom the vilest slanders and most shameful insinuations have been heaped from reputable and disreputable sources? His warmest friends admit the time has come to *speak out* and deny, or investigate the charges.

How did Infidelity in France and this country *first* wield the press for the rapid dissemination of false religion and unbridled license, by scattering leaves, tracts and newspaper articles broad cast, and how soon did the religious world seize the same agency to disseminate truth and purity wherever the art of printing was known.

We have hardly been honest to ourselves in this matter. We boast of having no secrets to conceal; our best attainments and most wonderful results, our choicest remedies, and most approved formulæ we never attempt to hide from the public; whoever enters the profession has access to all the grand results of generations gone before. We have no *blue* book or *cabalistic* signs, known only to the initiated. Our whole Science is open to any intelligent mind, and we have always been ready to give a reason for the medical faith within us to both *sick* and *well*; but as a profession we have been inclined to put a ban on all who proposed to instruct the Public. We have severely censured the display of successful practice either in medicine or surgery, we have even questioned the propriety of *Specialities.* We have been jealous of those, to whom ourselves and the public conceded special accomplishments.

We have kept in the ruts of preconceived prejudice, and educated bigotry far beyond any other *learned* profession. The Pulpit does not hesitate, while it *publicly* appeals to the masses on the most accessible rostrum in the land, to spread far and near old and new truths through the Press.

The Lawyer is ever advertising his wares at the most prominent and public locality in the County. He is always before the people in the Court-room, on the political rostrum and in the press; every judicial opinion is published in the press as well as in their official organs; and *why* should the *medical* profession be enforced to put their "light under a bushel," and muzzle their lips and their pens, while *ignorance, empiricism* and *pretension* boldly arrogate to themselves, knowledge, science and philanthropy by their garbled exhibitions in public, in their itinerant mountebanks; by their brazen assurance of *success* and *progress* on the public corners, in the press, by cards, hand-bills, Insurance Companies, divinity affidavits, and every other conceivable and inconceivable way?

Why these things should be, and *our* practice and ethics require of us a false modesty and an inappropriate silence, I do not understand. In fact under the head of "duties of the profession to the public;" (Code of Ethics) we are instructed to "be ever ready to give counsel to the public, in relation to matters especially appertaining to our profession, as on subjects of medical police, public hygiene, and legal medicine." What farther charter do we want? It is our *practice* more than our ethics perhaps at fault. If Science is truth, publish it. If our profession is meritorious and right, it need not fear to enter the lists and challenge the utmost scrutiny. If it is as beneficient, noble and fine as the fathers and founders contended and meant it should be, why not enlist the Press, and every other popular agency we possess to plant it in the hearts of the people, and show them the true way? Why not rise in our might and put out these false lights, which have been held up to the people and which they have ignorantly followed?

They look to us as their natural guardians and protectors, and justly so, while we fail to recognize our responsibility and allow them to perish. *We* are more to blame than they. It is our business to give them light, to warn, instruct and encourage. But under *false* views, and wrong precedents, we fail to give the note of warning, and they are left, the legitimate prey of every noisy pretender and unprincipled and ignorant charlatan.

Let me read you a specimen, cut from a Kansas Paper of last month signed "Exchange," evidently doing good duty for the H. S.

### A FAMILY DOCTOR.

The oldest medical practice is that of the family. Long before doctors or even priests were intrusted with the care of the sick, the parent performed that office, and to this day, the first and often most important medicine is given by the head of the family. The father or mother first notices the invasion of disease, the headache, chill, pain or hoarse cough, or other symptoms which indicate the presence of the enemy, and they too give the first medicine. Now to do this wisely and successfully, is to arrest the disease and save the patient. If unskilfully or at random, you not only do not benefit but injure. Humpherey's Homeopathic Specifics are intended to meet this want: In each family case is a book of directions, which fully instructs the parent as to what the disease is, provide the remedy for it, and show how to give it. With such a simple case, costing but a few dollars, a family is provided for emergencies, and will save largely in sickness, suffering and expense. —*Exchange.*

The address of HUMPHEREY'S HOMEOPATHIC MEDICINE CO. is 502 Broadway, N. Y.

Thus does the "*ignis fatuus*" of infinitesimals cajole the public, and with a few grains of general truth, attempt to conceal the fatal hook of *nihilism.*

Because *toleration* is right is it in the ascendant? Because *equality* in civil rights is recognized by the Constitution have the *people* so accepted it? No reform or advance can be made without a healthy public sentiment, and this public sentiment must be organized to be efficient. You cannot sell *coal* for *diamond,* though the elements may be there.

We number *ten* to *one* of our enemies, but they have captured the Press and by their persistent noise and bluster confuse the public and paralyze the truth.

We have County, District and State organizations, but we are hedged in by such an oppressive sense of our dignity, such solicitude for our position and Ethics, such exclusiveness for our professional rights and decorum, that we have not given *legitimate* publicity to much of our labor and practice, thereby depriving ourselves of public sympathy and confidence. We have failed to influence the legislation of the Country. We have not even secured the privilege of dissecting the worst criminal or any other cadaver legitimately, and yet every year bears witness to some suit of Mal-Practice for want of this very knowledge, which legislation requires and denys.

We see unsuspecting and innocent invalids all over our Country made bankrupt, plundered, robbed and left to perish by the most rascally Medical Shysters,—unprincipled humbugs and designing quacks, and no voice lifted, or efforts made, to protect life or property. Farmers can rise like a *Nemesis* in their power and grandure and change the whole course of legislation, seize the judiciary, and give law and enforce it too, in a single season, just by a little co-operation, or quiet organization; while we "*dummies*" sit silent, see humanity crippled, crushed and butchered, sent to their long home by false medicine and *doctrine;*—know how to arrest it, but confirmed fatalists, mumbling "what is to be, will be,"—never utter a protest, or give any alarm of danger. It is time this state of things was brought to an end. But how? Shall we violate our Ethics? Shall we secede? Shall we put ourselves outside of the pale of sympathy with the profession? Shall we go over to the enemy, and become irregular? No! none, nor either.

The National Association has endorsed by its silence the recommendation of its presiding officer, and *now* is the propitious time to agitate, yes; *agitate* is the word, agitate in our own boundaries the inalienable rights of man and the profession. Flash the bright light of truth on the public mind, through the daily press; iterate and reiterate the gross, glaring wickedness practiced on the simple, ignorant and unsuspecting by these *itenerant* sharks, *half educated* home doctors and the crowd of medical shysters and pretenders.

We have the *ability*, if we had the *will;* and every community has enough facts and figures if properly arranged and presented to make their ears tingle and face crimson. We can better shape public *medical* sentiment, than lawyers can the *political*, or clergymen the *theological*, for we number more and have better access to the masses, and it is from sheer neglect we have allowed such a false state of things to exist; we have *slept*, while the enemy has sowed tares.

But you say a mountain of ignorance and prejudice is to be overcome; very well, let it stimulate you the more; it is largely the result of your *own* neglect. Throw off this lethargy; arouse to duty! surround yourself with the best lights on Physiology and Hygiene and place facts and figures before the public;—tell them, how *proper* living will ensure good health,—what will protect from contagion—what to do and how to guard against endemic and epidemic disease. Paint in full colors the abuse of liquor, wine, beer, tea, coffee and tobacco. Teach them the effect of *sudden* changes, the necessity of appropriate clothing—diet—air, exercise and bathing. All these, the masses know but little about. Selections from our Journals could be given, imparting useful information· Take for instance the last *Medical Examiner* of June 15th, an extract from the *Medical and Surgical Reporter* "Is *Hard* Water wholesome?" demonstrating briefly, that it is much more wholesome than *soft* water. The influence of *locality, season* and a thousand other items, coming before us, could be compressed into a seasonable and entertaining article at least every month,—a taste and relish would thus be created, agreeable to publisher and reader, and the people gradually educated to *right* views, and placed on their guard against the false and dangerous. Will you do it? or will you sleep on, regardless of the life and happiness of many whom you love?

If these views are false, prove them so, and show us a better way, if true, awake to duty, cry aloud—and spare not, endorse them by action. If you know a community or an individual to be in danger, you commit a criminal act if you neglect to warn them; you are the custodians of their health, and greatly responsible for their moral, mental and physical obliquities. Will you give the alarm? or let them go, where hope and health can never reach them?

(NOTE.)—Since this paper was read, we find the following by the President of the Ohio State Medical Society, published in the "Clinic" of June 21st.

It seems strange to me, that some of our enterprising Journalists, instead of having some half dozen or more reporters employed to record the trivialities of every day life, do not employ at least one eminent medical writer, whose duty it shall be to set forth clearly and simply the best means of husbanding the vigor of youth for the weakness of old age, for criticising closely practitioners in medicine; thoroughly ventilating traveling imposters; to begin a warfare against patent nostrums.

* * * * * * * * * * * * As Physicians and philanthropists we should not be content to simply denouuce such a curse to the afflicted, but more, to look upon the most direct means to reach the sufferers, who are day by day poisoning themselves.

To this end a column of a single popular daily paper would do more good than all the medical journals combined."

